# RhoA regulates resistance to irinotecan by regulating membrane transporter and apoptosis signaling in colorectal cancer

**DOI:** 10.18632/oncotarget.13548

**Published:** 2016-11-24

**Authors:** Huang Ruihua, Zhang Mengyi, Zhao Chong, Qiu Meng, Ma Xin, Tang Qiulin, Bi Feng, Liu Ming

**Affiliations:** ^1^ Department of Medical Oncology/Laboratory of Signal Transduction and Molecular Targeted Therapy, West China Hospital, Sichuan University, Chengdu, Sichuan Province, China; ^2^ Department of Radiotherapy, The Tumor Hospital of Chengdu/The Seventh Peoples's Hospital of Chengdu, Chengdu, Sichuan Province, China

**Keywords:** irinotecan, RhoA, colorectal cancer, chemoresistance

## Abstract

Colorectal cancer (CRC) is a major cause of mortality and morbidity worldwide. While surgery remains the mainstay of treatment in early stage CRC, chemotherapy is usually given to prolong the overall survival and improve the quality of life for metastatic colorectal cancer (mCRC). But drug resistance is one of the major hurdles of mCRC treatment, and the underlying mechanisms are still largely unknown. In this study, we show that, compared with parental cells, RhoA is up-regulated in irinotecan (CPT-11)-resistant CRC cells. Furthermore, inhibition of RhoA in drug resistant cells, at least partially, rescues the resistance against irinotecan and increases the sensitivity to other chemotherapeutic drug by inhibiting expression of MDR1, MRP1and GSTP1, promotes apoptosis by suppressing the expression of BCL-XL and Bcl-2 and increasing Bax expression, and significantly decreases side population cells. Our results suggest that, in addition to survival, proliferation, migration, adhesion, cell cycle and gene transcription, RhoA is also involved in chemoresistance by regulating the expression of membrane transporter and apoptosis protein in colorectal cancer. They raise an interesting possibility that the expression of RhoA may indicate a poor prognosis due to the high probability to therapy resistance and, on the other hand, inhibition of RhoA activity and function may overcome chemoresistance and improve the effectiveness of clinical treatment of CRC.

## INTRODUCTION

Colorectal cancer is one of the most malignant cancers of digestive system, and its morbidity is increasing with years. The main treatment at present is surgery- plus chemotherapy-based comprehensive therapy. Irinotecan is one of common chemotherapeutic drugs for colorectal cancer, which inhibits DNA synthesis of cancer cells through inhibition of Topo I activity [1–3]. At present, FOLFIRI regimen, including CPT-11, 5-Fu and leucovorin, was considered as one of the standard first line or second line treatment of mCRC [4]. However, most patients become resistant to chemotherapy after a couple of cycles, leading to the failure of treatment. Therefore, chemoresistance is a bottleneck constraint of current therapies and the effectiveness has been seriously hindered. The mechanisms of chemoresistance in cancer cells are complicated and main mechanisms include (1) increased drug efflux mediated by membrane transport proteins such as ABC transport proteins family members p-glycoprotein (p-gp), multidrug resistance-associated protein (MRP), and breast cancer resistance protein (BCRP/ABCG2) [5, 6, 12]; (2) reduction in cell apoptosis mediated by bcl-2 family [7–9], NF-kB, and p53 [10]; and (3) other drug resistance mechanisms mediated by topoisomerase and glutathione S-transferase (GST). The reduction of topoisomerase activity leads to decreased affinity of topoisomerase with its target cells, which rendering target cells resistant to chemotherapy [11].

Rho GTPases family is an important intracellular signaling module and plays a key role in cell survival, proliferation, adhesion, cell cycle, gene transcription and other aspects of cell activities. While a subset of Rho GTPases are constitutively active, the majority of Rho family members act as molecular switches, cycling between the active, GTP-bound form and the inactive, GDP-bound form that are controlled by guanine nucleotide exchange factors (GEFs), GTPase-activating proteins (GAPs) and GDP-dissociation inhibitors (GDIs) [13]. As a primary Rho GTPase, RhoA is involved in multiple aspects of tumor development and progression, such as proliferation, migration, invasion, metastasis, and apoptosis [14, 15]. However, whether and how RhoA regulates drug resistance in colorectal cancer are poorly understood.

CPT-11 is one of the key chemotherapy agents for mCRC and resistance to CPT-11 is a major reason for the treatment failure. We established the CPT-11-resistant cells by exposure to a low dose of CPT-11 in culture media for 12 months, and preliminary results of comparative proteomic analysis of CPT-11-resistant CRC cells and parental cells show that irinotecan resistance is related to changes in several pathways such as cell proliferation/differentiation, cell apoptosis, and electron transport/redox regulation [16, 17]. In this study, we have explored the role of RhoA on CPT-11 resistance and underlying molecular mechanisms. Our study has provided a novel insight into the molecular mechanisms of drug resistance in colorectal cancer, and suggested that targeting RhoA signaling might improve the effectiveness of clinical treatment of colorectal cancer.

## RESULTS

### RhoA expression is increased significantly in CPT-11-resistant CRC cells

**Figure 1:**
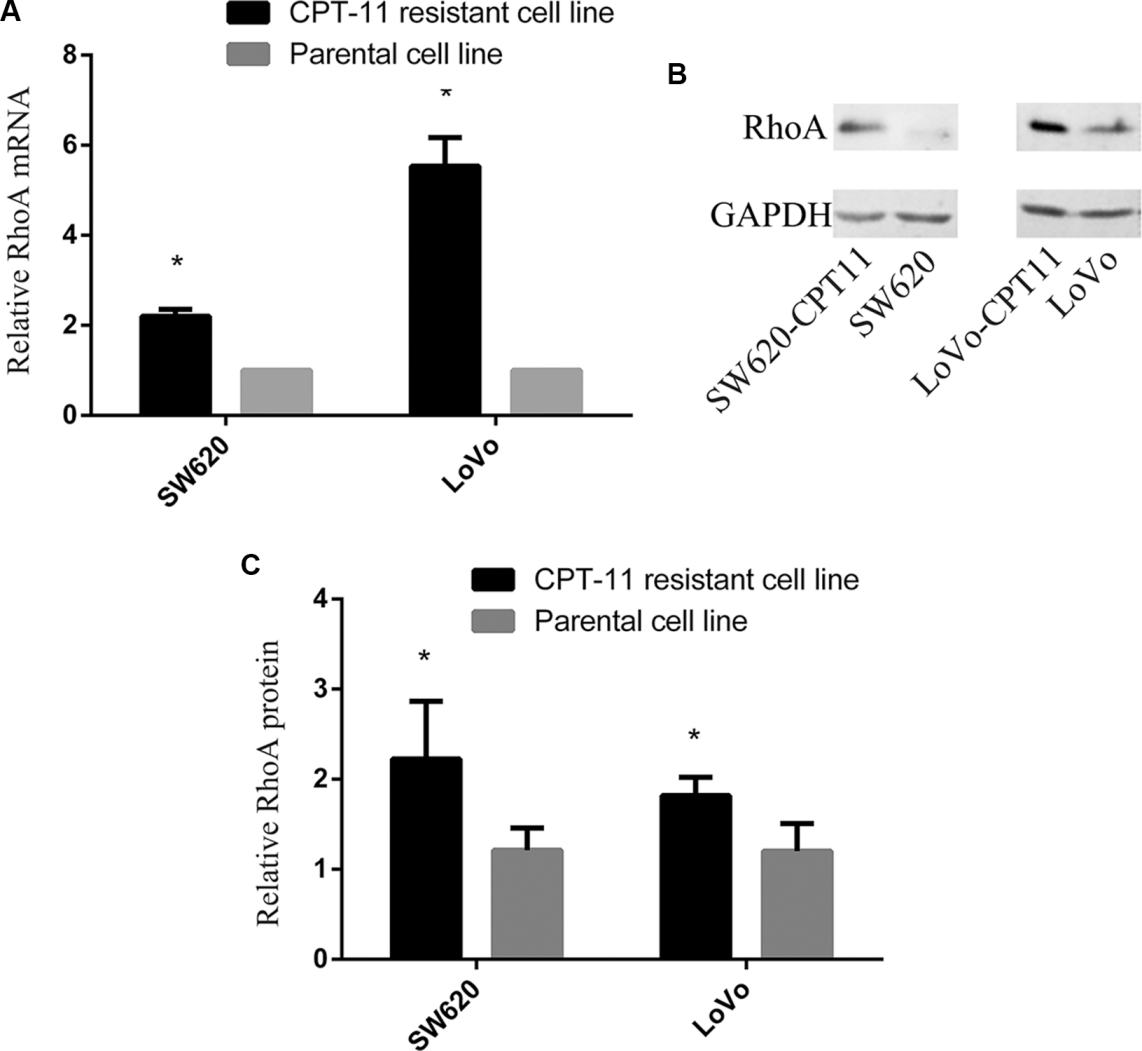
RhoA expression is increased significantly in CPT-11-resistant CRC cells (**A**) qRT-PCR analysis of RhoA mRNA expression in CPT-11-resistant cells and parental cells. RhoA mRNA expression was normalized to GAPDH mRNA expression, and data are shown as a ratio of CPT-11-resistant cells to parental cells using the 2-^−ΔΔCT^ method. (**B**) Western blot analysis of SW620/CPT-11, LoVo/CPT-11 and parental cells for RhoA protein expression. (**C**) The band intensities in B were quantified with the ODYSSEY Infrared Imaging System (LI-COR Biosciences). Data represent the mean ± S.D. from three independent experiments. *indicates *p <* 0.05.

To determine if RhoA is involved in chemoresistance in colorectal cancer, we first examined the expression of RhoA in CPT-11-resistant colorectal cancer cells (SW620/CPT-11 and LoVo/CPT-11) and parental cells. Total mRNAs and proteins of SW620/CPT-11, LoVo/CPT-11 and its parental cells were extracted and the expression of RhoA mRNA and protein were analyzed by qRT-PCR and Western blot, respectively. The results showed that RhoA mRNA and protein expression levels were higher in SW620/CPT-11 and LoVo/CPT-11 than in parental cells (Figure 1). These results suggest a possible relationship between RhoA and chemoresistance in CRC.

### Inhibition of RhoA expression, at least partially, rescues CPT-11 chemoresistance of CRC cells

To examine if the increased RhoA expression is important for chemoresistance of CRC, we transfected SW620/CPT-11, LoVo/CPT-11 and parental cells with siRNA targeting RhoA coding sequences (si-RhoA) and control siRNA oligo (si-control), and then examined drug resistance index of the cells to oxaliplatin (L-OHP), cisplatin(DDP), Irinotecan (CPT-11), paclitaxel (PTX), 5-FU, epirubicin (EPI), and Etoposide (VP-16), by *in vitro* drug sensitivity assay. As shown in Table 1, the IC50 of all of the 7 chemotherapy drugs in CPT-11-resistant CRC cells was significantly increased compared to that in parental cells. However, the IC50 was significantly decreased upon transfection of si-RhoA (*p <* 0.05). These data suggest that inhibition of RhoA expression in CPT-11-resistant CRC cells, at least partially, overcomes chemoresistance of CRC.

**Table 1:** Inhibition of RhoA expression, at least partially, rescues chemoresistance of CRC cells

	CPT-11	5-FU	PTX	DDP	EPI	OHP	VP16
**SW620**	0.58 ± 0.04	0.50 ± 0.08	0.15 ± 0.02	0.12 ± 0.05	0.42 ± 0.04	1.07 ± 0.15	0.15 ± 0.12
**SW620/CPT-11**	12.91 ± 0.79*	1.31 ± 0.21*	1.17 ± 0.20*	0.79 ± 0.35*	2.33 ± 0.10*	4.08 ± 0.06*	1.87 ± 0.26*
**Si-control**	12.31 ± 0.89	1.23 ± 0.21	1.23 ± 0.13	0.77 ± 0.25	2.04 ± 0.04	4.04 ± 0.04	1.84 ± 0.21
**Si-RhoA**	3.68 ± 0.17*	0.87 ± 0.06*	0.35 ± 0.04*	0.23 ± 0.03*	0.46 ± 0.01*	1.9 ± 0.07*	0.29 ± 0.03*
**LoVo**	1.83 ± 0.33	0.24 ± 0.03	0.14 ± 0.03	0.11 ± 0.02	0.35 ± 0.05	0.57 ± 0.04	0.24 ± 0.03
**LoVo/CPT-11**	15.57 ± 1.43*	6.04 ± 0.54*	1.07 ± 0.22*	1.47 ± 0.09*	1.45 ± 0.49*	3.27 ± 0.32*	1.50 ± 0.29*
**Si-control**	14.95 ± 0.10	5.39 ± 0.67	1.07 ± 0.03	1.55 ± 0.15	1.22 ± 0.32	3.18 ± 0.21	1.51 ± 0.22
**Si-RhoA**	4.69 ± 0.30*	0.30 ± 0.01*	0.74 ± 0.06*	0.44 ± 0.02*	0.72 ± 0.10*	0.55 ± 0.10*	0.29 ± 0.04*

SW620/CPT-11, LoVo/CPT-11 and parental cells were transfected with si-RhoA or si-control, and then drug resistance to L-OHP, DDP, CPT-11, PTX, 5-FU, EPI and VP-16 was tested by *in vitro* drug sensitivity assay. Data are representative of three independent experiments performed in triplicate.

* indicates a significant difference from control oligo-transfected cells (*p <* 0.05).

### Inhibition of RhoA suppresses the expression of P-gp, MRP1 and GSTP1 in CPT-11-resistant CRC cells

**Figure 2:**
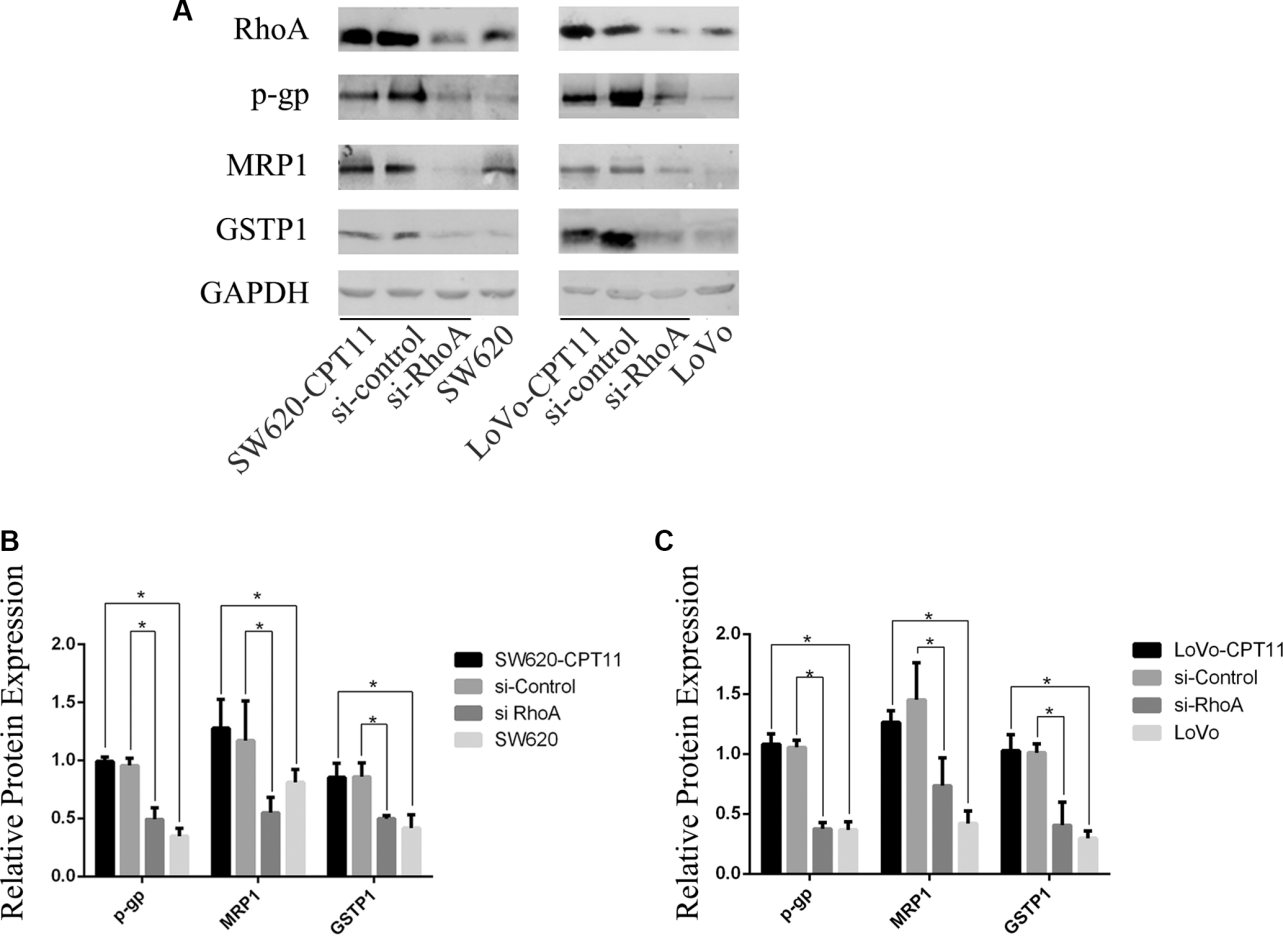
Inhibition of RhoA suppresses the expression of P-gp, MRP1 and GSTP1 in CPT-11-resistant CRC cells (**A**) Western blot analysis of CPT-11-resistant CRC cells (SW620/CPT-11, LoVo/CPT-11), CPT-11-resistant CRC cells transfected with si-control or si-RhoA, and parental cells for RhoA, P-gp, MRP1 and GSTP1 protein expression. (**B**) and (**C**) The band intensities in A were quantified with the ODYSSEY Infrared Imaging System (LI-COR Biosciences). Data represent the mean ± S.D. from three independent experiments. *indicates *p <* 0.05.

To explore the mechanisms underlying RhoA-regulated chemoreistance, Western blot was performed to determine the expression of p-glycoprotein(P-gp) and multidrug resistance-associated protein 1(MRP1), two membrane transport proteins known to mediate drug efflux, in CPT-11-resistant CRC cells and parental cells. As expected, P-gp and MRP1 were up-regulated in CPT-11-resistant CRC cells (Figure 2). Next, to test whether P-gp and MRP1 are regulated by RhoA, SW620/CPT-11 and LoVo/CPT-11 cells were transfected with si-RhoA or control, and expression of P-gp and MRP1 were examined 48 hours after transfection. Interestingly, inhibition of RhoA by si-RhoA suppressed P-gp (Figure 2A–2C) and MRP1(Figure 2A–2C) expression in CPT-11-resistant CRC cells.

In addition to membrane transporting proteins, glutathione S-transferase(GST) is also involved in drug resistance [18]. The expression of GSTP1, an important member of GST family, was determined by Western blot in CPT-11-resistant CRC cells and parental cells. As shown in Figure 2, GSTP1 was up-regulated in CPT-11-resistant CRC cells and inhibition of RhoA by si-RhoA suppressed GSTP1 expression (Figure 2A–2C). These data suggest that both of membrane transport proteins and GST are regulated by RhoA, which, at least partially, contribute to CPT-11 resistance of CRC cells.

### Inhibition of RhoA induces apoptosis in CPT-11-resistant CRC cells

**Figure 3:**
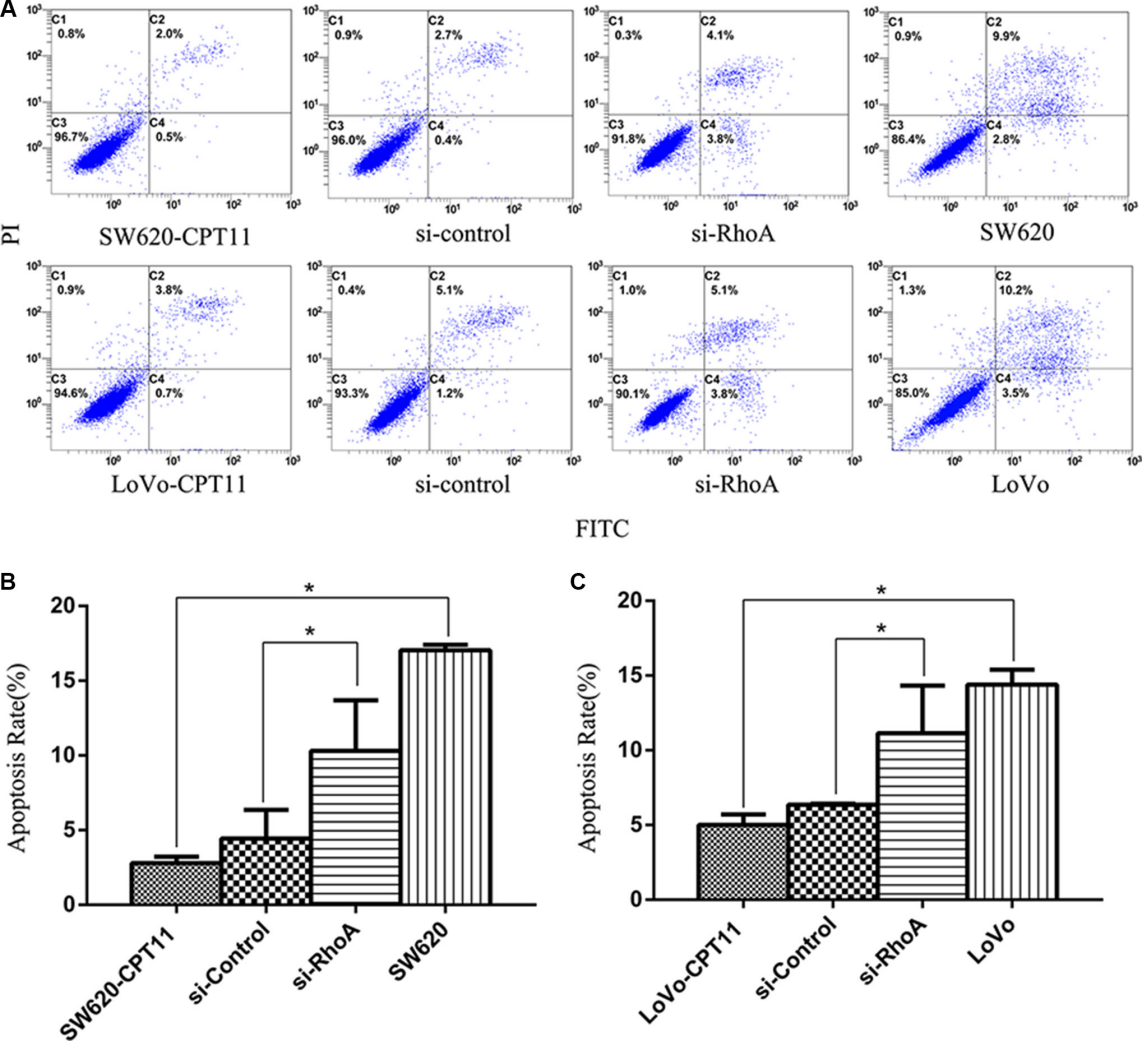
Inhibition of RhoA induces apoptosis in CPT-11-resistant CRC cells by FACS analysis (**A**) The apoptosis rates of CPT-11-resistant CRC cells (SW620/CPT-11or LoVo/CPT-11), CPT-11-resistant CRC cells transfected with si-control or si-RhoA, and parental cells were determined by FACS analysis. (**B**) and (**C**) Apoptosis rate of CRC cells in A was quantified. Data are representative of three independent experiments. *indicates *p <* 0.05.

**Figure 4:**
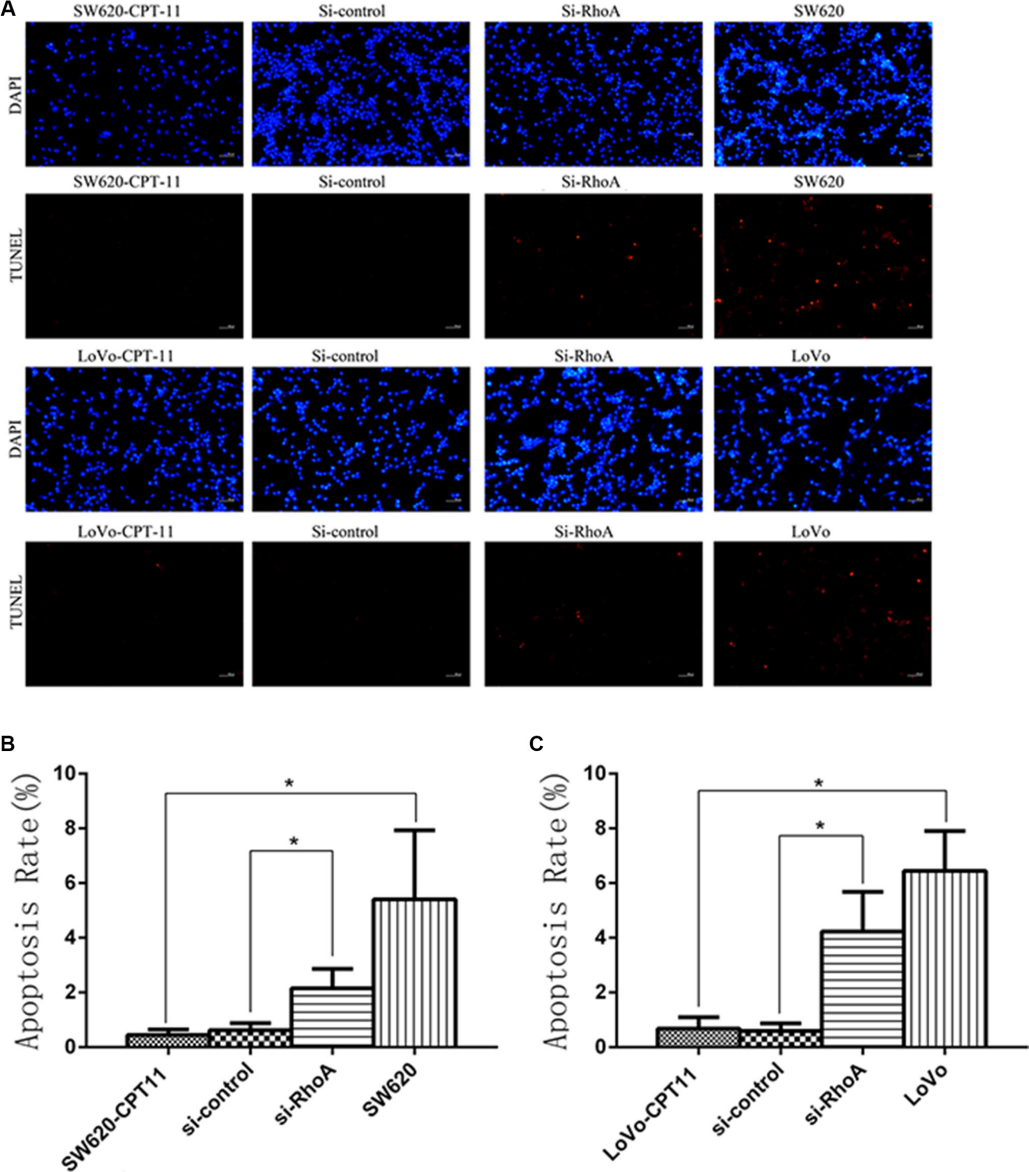
Inhibition of RhoA induces apoptosis in CPT-11-resistant CRC cells by TUNEL assay (**A**) The apoptosis rates of CPT-11-resistant CRC cells (SW620/CPT-11or LoVo/CPT-11), CPT-11-resistant CRC cells transfected with si-control or si-RhoA, and parental cells were determined by TUNEL assay. (**B**) and (**C**) Apoptosis rate of CRC cells in A was quantified. Data are representative of three independent experiments. *indicates *p <* 0.05.

To further understand the mechanisms of RhoA in the regulation of chemoresistance of CRC cells, we investigated the effects of RhoA on apoptosis in CPT-11-resistant CRC cells, as evasion of apoptosis is a crucial event during the process of chemoresistance. We found that, compared with parental cells, CPT-11-resistant CRC cells showed a significant decrease in apoptosis rate, as determined by Annexin V-FITC/PI staining (Figure 3A–3C), whereas inhibition of RhoA by si-RhoA resulted in a significant increase in apoptosis rate in CPT-11-resistant CRC cells (Figure 3A–3C). Similar results were obtained by TUNEL assay (Figure 4A–4C). These results suggest that inhibition of RhoA induces apoptosis in CPT-11-resistant CRC cells.

**Figure 5:**
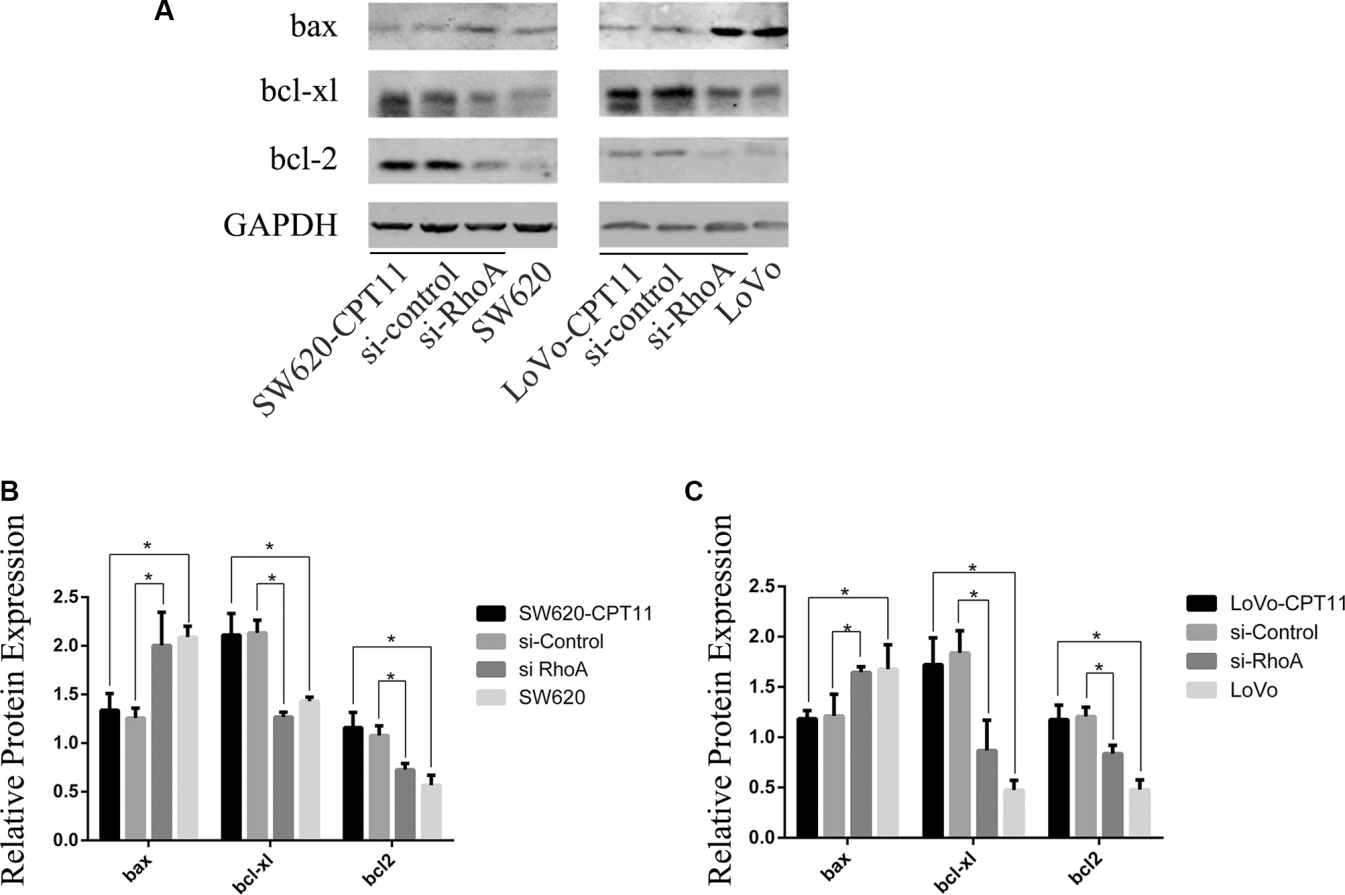
RhoA affects the expression of bcl-2, bcl-xl and bax in CPT-11-resistant CRC cells (**A**) Western-blot analysis of CPT-11-resistant CRC cells (SW620/CPT-11or LoVo/CPT-11), CPT-11-resistant CRC cells transfected with si-control or si-RhoA, and parental cells for bcl-2, bcl-xl and bax protein expression. (**B**) The band intensities in A were quantified with the ODYSSEY Infrared Imaging System (LI-COR Biosciences). Data represent the mean ± S.D. from three independent experiments. *indicates *p <* 0.05.

To characterize the mechanistic role of RhoA in apoptosis in CPT-11-resistant CRC cells, Western blot was performed to determine the protein expression of bcl2 family members in CPT-11-resistant CRC cells and parental cells. As shown in Figure 5, pro-apoptotic bax was down-regulated, and anti-apoptotic Bcl-xl and Bcl2 were up-regulated in CPT-11-resistant CRC cells. To determine whether bax, Bcl-xl and Bcl2 are regulated by RhoA, SW620/CPT-11 and LoVo/CPT-11 cells were transfected with si-RhoA, and expression of bax, Bcl-xl, and Bcl2 were examined by Western blot 48 hours after transfection. Interestingly, inhibition of RhoA by si-RhoA suppressed the expression of Bcl-xl and Bcl2, and promoted the expression of bax in CPT-11-resistant CRC cells (Figure 5A–5C). These results suggest that CPT-11 chemoresistance of CRC cells could be attributed to the decreased apoptosis that is regulated by RhoA through regulating Bcl-2 signaling.

### Inhibition of RhoA decreases the proportion of SP cells in CPT-11-resistant CRC cells

**Figure 6:**
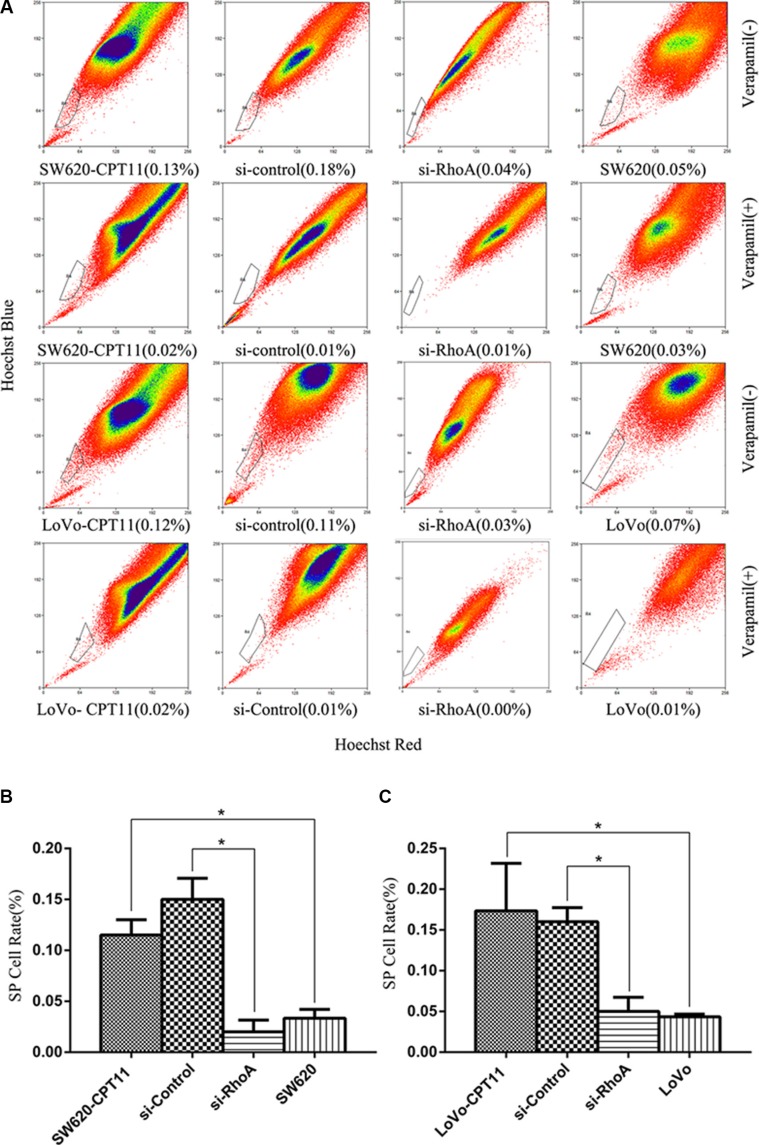
Inhibition of RhoA decreases the proportion of SP cells in CPT-11-resistant CRC cells (**A**) The proportion of SP cells was detected using the Hoechst-33342 dye exclusion technique in CPT-11-resistant CRC cells (SW620/CPT-11or LoVo/CPT-11) and CPT-11-resistant CRC cells transfected with si-control or si-RhoA, and parental cells. (**B**) and (**C**) The proportion of SP cells in A was quantified (*p <* 0.05). Data are representative of three independent experiments. *indicates *p <* 0.05.

Several studies have demonstrated that isolated side population (SP) cells from solid tumors exhibit cancer stem cell-like properties, and are responsible for drug resistance during chemotherapy and tumor recurrence [19]. To investigate the role of SP cells in CPT-11 resistance of CRC cells, and the role of RhoA in SP cells in CPT-11-resistant CRC cells, we detected the proportion of SP cells using the Hoechst/33342 dye exclusion technique in CPT-11-resistant CRC cells and parental cells. Compared with parental cells, the proportion of SP cells were increased significantly in both of the CPT-11-resistant CRC cells (SW620-CPT11,LoVo-CPT11) (*p <* 0.05, Figure 6A–6C), whereas suppression of RhoA led to a drastic decrease of SP cells in CPT-11-resistant CRC cells (*p <* 0.05, Figure 6A–6C). These data suggest that RhoA-induced SP cells contribute to drug resistance of CRC cells.

## DISCUSSION

CRC is a major cause of mortality and morbidity worldwide [1]. For the advanced CRC, chemotherapy is recommended as a major treatment. But drug resistance highly limits therapeutic options and its molecular mechanism needs to be understood urgently. As a key member of the Rho GTPase family, RhoA is known to be involved in proliferation, migration, invasion, apoptosis, and angiogenesis, in several types of cancers [13–15]. However, the role of RhoA in chemoresistance is poorly defined. In this study, we investigated the effects of RhoA on CPT-11 resistance and underlying molecular mechanisms in detail. The results suggest that increased expression of RhoA promotes resistance to irinotecan by regulating membrane transporter and apoptosis signaling in CPT-11-resistanct CRC cells. Our findings are in line with that by Carloni V who has shown that RhoA is involved in cell fusion and causes the appearance of cells resistant to both 5-fluorouracil and oxaliplatin in a metastatic model of colon carcinoma [28]. Thus, the expression of RhoA may indicate a poor prognosis due to the high probability to therapy resistance.

ABC transporters transport a wide range of substrates including metabolic products, nutrients, lipids, and drugs across extracellular and intracellular membranes. ABC transporters are well-known to be associated with chemoresistance. Expression of various ABC transporters is increased following chemotherapy, causing a reduction in the intracellular accumulation of chemotherapy drugs, and impacting on mCRC survival [12]. In support, our study found increased expression of ABC transporters, MRP1 and P-gp, in CPT-11-resistant CRC cells. Importantly, suppression of RhoA in CPT-11-resistant CRC cells leads to decreased expression of MRP1 and P-gp. We propose that inhibition of RhoA could rescue chemoresistance, at least partially, by dampening ABC transporter expression in CRC cells.

In addition to increased ABC transporters, we have further found that suppression of apoptosis is another important mechanism of drug resistance of CRC and that RhoA critically inhibits apoptosis in CPT-11-resistant CRC. Studies have shown that Bcl-2 and Bax play important roles in mediating drug-induced apoptosis and drug resistance in various tumor cells, including hepatocellular carcinoma, bladder, lung and ovarian cancer [20–23]. Here we show that RhoA inhibits drug-induced apoptosis by impacting on Bcl-2, Bcl-xl and Bax.

Growing evidences support the notion that cancer is a disease driven by cancel stem cells that are responsible for tumor initiation, growth, metastasis, therapy resistance, relapse, and poor prognosis [24–27]. Several studies have demonstrated that the isolated side population (SP) cells from solid tumors exhibit cancer stem cell-like properties, and are responsible for drug resistance during chemotherapy and tumor recurrence [24–27]. In the current study, we have found that SP cells are increased in CPT-11-resistant CRC cells and suppression of RhoA leads to a sharp decrease of SP cells. It indicates that, in addition to membrane transport proteins and survival, cancer stem cells/SP cells are also regulated by RhoA and contribute to drug resistance of CRC.

In conclusion, our study shows that RhoA is involved in chemoresistance of CRC by regulating the expression of membrane transporters, apoptotic proteins and the proportion of SP cells. Thus, the expression of RhoA may indicate a poor prognosis due to the high probability to therapy resistance and, on the other hand, chemoresistance can be compromised through interfering RhoA expression.

## MATERIALS AND METHODS

### Cell culture

All cell lines were maintained in RPMI 1640 medium (Invitrogen, Gaithersburg, MD, USA) supplemented with 10% heat-inactivated fetal bovine serum (FBS) (Gibco, Gaithersburg, USA), 100 units/ml of penicillin G sodium, and 100 μg/ml streptomycin sulfate (Sigma, Saint Louis, MO, USA), in a humidified atmosphere containing 5% CO_2_ at 37°C.

### RNA extraction and qRT-PCR analysis

Total RNA was extracted using Trizol (Invitrogen), treated with DNase I (Takara) to eliminate contaminating genomic DNA, and reverse-transcribed into cDNA with the Reverse Transcriptase M-MLV (TaKaRa). Real time PCR was performed using a SYBR Premix Ex TaqTM kit (TaKaRa) on the iQ5 Real-Time PCR Detection System (Bio-Rad, Hercules, CA, USA). PCR primers used were as following: GAPDH FW, 5′-GAAGGTGAAGGTCGGAGT-3′ and RV 5′-GAAGAT GGTGATGGGATTTC-3′, RhoA FW 5′-GATTGGCGCT TTTGGGTACAT-3′ and RV 5′-AGCAGCTCTCGTA GCCATTTC-3′. Expression of RhoA, relative to GAPDH, was determined using the 2^−ΔΔCT^ method.

### Western blot analysis

Total-cell lysates were prepared using RIPA buffer (150 mM NaCl, 1%NP-40, 50 mM Tris-HCl (pH 7.4), 1 mM phenylmethylsulfonyl fluoride, 1 μg/ml leupeptin, 1 mM Deoxycholic acid and 1 mM EDTA) containing a cocktail of protease inhibitors and phosphatase inhibitors (Calbiochem, Darmstadt, Germany). Equal amount of proteins (40∼60 μg) was separated by 12% SDS-PAGE and transferred to PVDF membrane (Millipore, Bedford, MA, USA) using the Bio-Rad semidry transfer system. The following antibodies were used for Western blot: Anti-RhoA antibody (Santa Cruz Biotechnologies, USA), Anti-GAPDH antibody, Anti-bcl2 antibody, Anti-bcl-xl antibody, Anti-bax antibody (Cell Signaling Technology, USA), Anti-ABCB1 antibody, Anti-ABCC1 antibody, Anti-GSPT1 antibody (Abcam, UK). Blotted proteins were detected and quantified using the ODYSSEY Infrared Imaging System (LI-COR Biosciences, Lincoln, NE, USA).

### Transfection

Control siRNA and RhoA-siRNA targeting RhoA coding sequences (5′-UGAGCAAGCAUGUCUUUCCAC AGGC-3′, 5′-GCCUGUGGAAAGACAUGCUUGCUCA-3′) were synthesized by RiboBio (RiboBio, Guangzhou, China). RNA oligonucleotides were transfected into cells using Lipofectamine 2000 (Invitrogen) according to the manufacturer's protocol.

### *In vitro* drug sensitivity assay

Cells were seeded onto 96-well culture plates at a density 1.0 × 10^3^/well with RPMI1640 100 ul per well. A total of 4 groups were set in this study: CPT-11 resistant group, siRNA control group, RhoA siRNA group, parental colorectal cancer cell group. Chemotherapeutic drugs CPT-11, L-OHP, PTX, VP16, EPI, DDP, 5-FU (Dalian Meilun Biology Technology co., LTD, China) were added at increasing concentration for 24 hours. The cells were continuously incubated for 48hours after removing the chemotherapeutic drugs. *In vitro* drug sensitivity was then determined using Cell Counting Kit-8 assay (Dojindo Molecular Technologies, Inc.Japan). In brief, the cells were added of 10 ul of CCK-8, incubated for three hours, measured for the absorbance at 450nm, and calculated for the inhibitory concentration of 50% cells (IC_50_). The assays were conducted in triplicate and repeated at least 3 times.

### Flow cytometry

1 × 10^6^ cells were collected by centrifugation and resuspended using 500 ul bonding buffer (KeyGen Biotechnology Co., Ltd., China). 5 μl of Annexin V-FITC and 5 μl of propidium iodide were added into the collected cells (KeyGen Biotechnology Co., Ltd., China) and incubated for 5 minutes at room temperature in the dark. Annexin V-FITC binding was analyzed by flow cytometry (Ex = 488 nm; Em = 530 nm) using FITC signal detector and PI staining by the phycoerythrin emission signal detector (Beckman Coulter, Inc., USA). The assays were conducted in triplicate and repeated at least 3 times.

### TUNEL assay

TUNEL assay is performed with a kit from Millipore according to the manufacturer's instructions and observed by a fluorescence microscopy (Nikon, Japan). The assays were conducted in triplicate and repeated at least 3 times.

### SP analysis

The cells were harvested and resuspended at 1 × 10^6^/ml in prewarmed DMEM (Life technologies, USA) containing 2% fetal calf serum (Kang Yuan Biology, China) and 10 mM HEPES (Dalian Meilun Biology Technology co., LTD, China). The cell suspension was gently mixed in 15 ml polypropylene centrifuge tube (Corning, USA). And then Hoechst 33342 (Invirtrogen, USA) was added to a final concentration of 5 ug/ml and verapamil (Sigma-Aldrich, USA) of 80 ug/ml to control group. The cells were incubated for 90 minutes at 37°C and mixed every 30 minutes. After incubation, the cells were centrifuged at 500 g for 5 minutes, and resuspended in cold HBSS. Propidium iodide (Sigma-Aldrich,USA) was added into each tube at concentration of 1 ug/ml to discriminate dead cells. In order to avoid the effluxion of Hoechst 33342, further tests were performed at 4°C. SP cells were tested using flow cytometry and analyzed using Summit 5.2 software (Beckman Coulter, Inc., USA). The assays were conducted in triplicate and repeated at least 3 times.

### Statistical analysis

IC_50_ was calculated using probit regression analysis. The results were reported as Mean ± SD and analyzed by student *t-test* or one way ANOVA by SPSS 18.0 analytic software. *P* values of < 0.05 were considered as statistical significant.

## Abbreviations

RhoA: ras homolog gene family, member A
si-RhoA: siRNA targeting RhoA coding sequences
mCRC: metastatic colorectal cancer
CPT-11: irinotecan
MDR1: multidrug resistance 1
MRP1: multi-drug resistant associate protein
L-OHP: oxaliplatin
DDP: cisplatin
PTX: paclitaxel
5-FU: 5-fluorouracil
EPI: epirubicin
VP-16: etoposide
